# Analysis on Gene Expression Profile in Oncospheres and Early Stage Metacestodes from *Echinococcus multilocularis*

**DOI:** 10.1371/journal.pntd.0004634

**Published:** 2016-04-19

**Authors:** Fuqiang Huang, Zhisheng Dang, Yutaka Suzuki, Terumi Horiuchi, Kinpei Yagi, Hirokazu Kouguchi, Takao Irie, Kyeongsoon Kim, Yuzaburo Oku

**Affiliations:** 1 Parasitology Laboratory, School of Veterinary Medicine, Faculty of Agriculture, Tottori University, Tottori, Japan; 2 The United Graduate School of Veterinary Science, Yamaguchi University, Yamaguchi, Japan; 3 National Institute of Parasitic Diseases, Chinese Center for Disease Control and Prevention, Key Laboratory of Parasite and Vector Biology of the Chinese Ministry of Health, WHO Collaborating Center for Malaria, Schistosomiasis and Filariasis, Shanghai, China; 4 Graduate School of Frontier Sciences, University of Tokyo, Chiba, Japan; 5 Department of Infectious Disease, Hokkaido Institute of Public Health, Sapporo, Hokkaido, Japan; The First Affiliated Hospital of Xinjiang Medical University, CHINA

## Abstract

Alveolar echinococcosis is a worldwide zoonosis of great public health concern. Analysis of genome data for *Echinococcus multilocularis* has identified antigen families that can be used in diagnostic assays and vaccine development. However, little gene expression data is available for antigens of the egg and early larval stages. To address this information gap, we used a Next-Generation Sequencing approach to investigate three different stages (non-activated and activated oncospheres, and early stage metacestodes) of *E*. *multilocularis* (Nemuro strain). Transcriptome data analysis revealed that some diagnostic antigen gp50 isoforms and the antigen Eg95 family dominated in activated oncospheres, and the antigen B family dominated in early stage metacestodes. Furthermore, heat shock proteins and antigen II/3 are constantly expressed in the three stages. The expression pattern of various known antigens in *E*. *multilocularis* may give fundamental information for choosing candidate genes used in diagnosis and vaccine development.

## Author Summary

Humans become infected with *E*. *multilocularis* through oral ingestion of the eggs. Untreated alveolar echinococcosis has a fatality rate of >90% in humans, causing great public health concern in the northern hemisphere. The genome databases of *E*. *multilocularis* have been recently published, but still there is no available data about transcriptomic profile datasets of the early larval stages (non-activated and activated oncospheres and immature metacestodes). In this study, the gene expression profile of *E*. *multilocularis* oncospheres and metacestodes were tested using the Next-Generation Sequencing (NGS) approach. The expression level of hundreds genes was significantly different between oncospheres and metacestodes. Some known tapeworm-specific antigen family like diagnostic antigen gp50, antigen Eg95, and antigen B are predominated in oncospheres and metacestodes, respectively. Furthermore, within the tapeworm-specific antigen family, some antigen subunits or variations were stage-specific expression. The identification of genes expression profile during the parasite development stages, especially the stage of oncospheres, will give fundamental information for choosing candidate genes used in vaccination and early diagnosis.

## Introduction

Alveolar echinococcosis (AE) is a worldwide zoonosis that is of great public health concern in the northern hemisphere. Eggs of the tapeworm, which are excreted by definitive hosts, foxes and dogs, present a risk for humans [1]. After oral ingestion of mature oncosphere-containing eggs, the oncospheres hatch in the small intestine of the intermediate host, and then migrate via the hepatic vein to the liver, where they form cyst masses and increasingly transform into multiple vesicles filled with fluid and protoscoleces [2]. The metacestodes are lined with a germinal layer and a laminated layer, which allow the parasite to escape the host immune response and transition to the chronic stage in the liver [3–4].

It has been proven that infections can be blocked at the egg and early larval stages by antibodies and complement-dependent mechanisms [5]. Furthermore, *in vitro* hatching and activation of oncosphere have been achieved, showing that oncosphere has an extended excretion apparatus and proteinases that may contribute to a considerable portion of the excreted proteins during the penetration process [6, 7, 8, 9]. The fact that the excretory/secretory proteins produced in the early (oncosphere) and chronic (metacestode) infectious stages by *E*. *multilocularis* can cause significant apoptosis or immature of the dendritic cells (DC) [10] suggests that the early infective stage of *E*. *multilocularis* is a strong inducer of tolerance in DC, which is most probably important for producing an immunosuppressive environment in the infection phase.

Immune response to larval *Echoinococcus* spp. infections has been divided into “establishment” and “established metacestode” phases [5, 11]. And it is thought that the parasite are more susceptible to immune attack during early stages of infections (“establishment” phase) [5, 11]. The immunogenic to the tested models of numbers of recombinant proteins are available. It was reported that vaccine Eg95, which is based on the recombinant protein cloned from mRNA from the oncosphere of *E*. *granulosus* and shown to be highly effective in vaccine trials of sheep and had induced a high level of protection (96–100%) for more than a year post-vaccination [12]. In addition, AgB [13, 14], EmY162 [15], P29 [16, 17], EgEF [18], Eg19 [19] and TSPs [2, 20], derived from the *Echinococcus* spp., exhibit strong immunogenic properties in tested model, respectively. Furthermore, secondary AE, in which homogenates of the larval parasite are intraperitoneally, intravenously or intrahepatically injected into the host animals, is widely used; however, it does not reproduce the early stages of parasite development that occurs during natural infection via oral ingestion of the eggs [21]. In addition, immunisation with *E*. *multilocularis* 14-3-3 protein protected intermediate hosts from primary but not secondary challenge infection with AE [22]. One study show that the parasite lesions in the liver of primary AE at 4 weeks post inoculation varied among the strains of mice and suggests that the resistance to the early stages of parasite infections, including parasite establishment in the liver, is genetically regulated[21].

Vaccination and early diagnosis are possible ways to control and prevent echinococcosis. Accurate immunodiagnosis of early infection requires highly specific and sensitive antigens. At present, little gene expression data has been published for egg and early larval stages. Thus, experiments on identifying antigens for use in immunodiagnostic assays is a crucial point in the improvement of the diagnostic tool and must be based on the developmental stage of the parasite.

The genome databases of *E*. *multilocularis* have recently become available [23], and using the draft antigen families of *E*. *multilocularis*, gene expression profiles for adult and mature metecestode can be predicted, but transcriptomic profile datasets of the early larval stages (non-activated and activated oncospheres and immature metacestodes) are still unavailable.

As for mentioned above and gain understanding of the gene expression patterns for diagnostic assay and vaccine design, we analyzed the transcriptomes of non-activated and activated oncospheres, 4-week metacestodes miniature vesicles (Primary AE) and metacestodes small vesicles cultivated *in vitro* (Secondary AE) to identify homologues of the various known antigens of tapeworms, especially *Echinococcus* spp.

## Materials and Methods

### Ethics statement

This study was carried out in strict accordance with the recommendations set out in the Guidelines for Animal Experimentation of the Japanese Association for Laboratory Animal Science, and the protocol for the animal experiments was approved by the ethics committee of the Hokkaido Institute of Public Health (permit number: K25-02).

### Preparation of parasite samples

*Echinococcus multilocularis* isolated in Hokkaido (Nemuro strain) was routinely maintained through a dog–cotton rat life cycle at the Hokkaido Institute of Public Health (Sapporo, Japan). Dogs were orally administered 5 × 10^5^ *E*. *multilocularis* protoscoleces and the infection was terminated 35–77 days postinfection by administering two tablets of Droncit[24].

#### Non-activated oncospheres (Nonc)

Feces were collected from experimentally infected dogs at 35 days postinfection. Eggs were isolated from feces by filtering by mesh, natural sedimentation and flotation with sugar solution. The isolated eggs were treated with 3% sodium hypochlorite for 20 mins for removal of the embryophore and sterilization. Non-activated oncospheres were collected at two times for biological replicates: September 2013 (sample, Nonc1) and December 2013 (sample, Nonc2).

#### Activated oncospheres (Aonc)

Techniques for activation of non-activated oncospheres were as previously described [6, 9]. Briefly, non-activated oncospheres were activated with 1% pancreatin (Nacalai Tesque, Inc.), 1% hog bile extract (MP Biomedicals, LLC) and 0.2% Na_2_CO_3_ in RPMI 1640 (Gibco) at 38°C for 20 mins, and then cultivated in RPMI 1640 with 10% fetal calf serum (Gibco) at 38°C for 24 h.

#### 4-week metacestodes miniature vesicles (4Wmet)

The DBA/2 mice were sacrificed after four weeks post oral infections with eggs and small lesions with early stage larvae were collected from the livers. The collected larvae were examined as 4-week metacestodes miniature vesicles (4Wmet).

#### Metacestodes small vesicles cultivated *in vitro* (Cmet)

**Fig 1:**
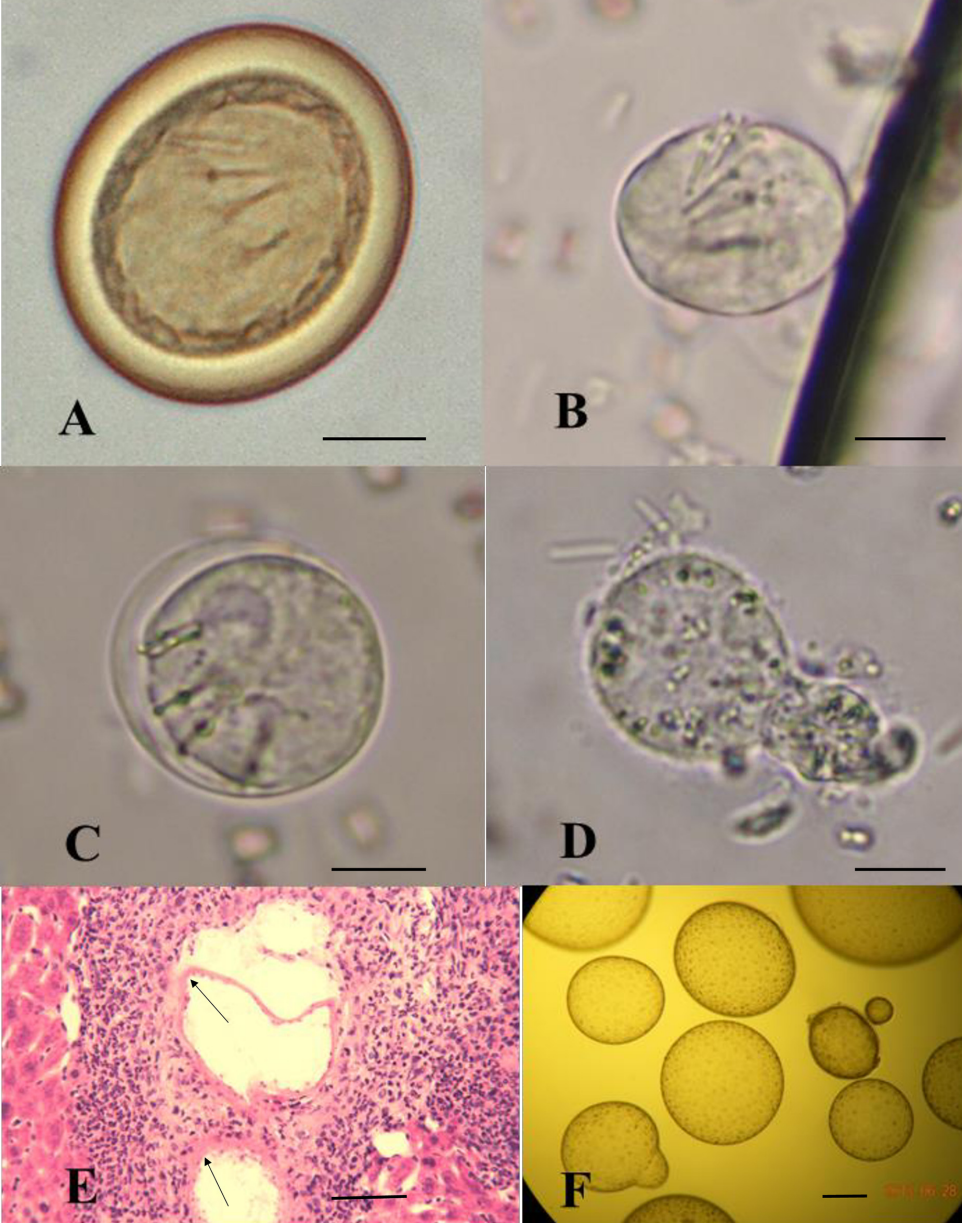
Morphology of different life cycle stages of *E*. *multilocularis*. A: Egg; B: Oncospheres (non-activated); C: Oncospheres (activating) with the hooks dispersive and body swelling; D: Oncospheres (activated) with the hooks aggregation in the smaller lobe after 24 hours activation; E: 4-week metacestodes miniature vesicles; F: Metacestodes small vesicles cultivated in vitro; Bar: 10μm (A-E), 1mm (F); Arrowhead: Miniature vesicles.

*In vitro* cultivation of *E*. *multilocularis* was carried out as described previously [25, 26]. In short, cyst masses of metacestodes from intraperitoneal passage DBA/2 mice at 16 weeks were cut into small pieces and cultivated in DMEM (Gibco) with 10% fetal calf serum (Gibco) at 37°C. Miniature cysts were grown to small vesicles (2–4 mm in diameter) in several weeks but were harvested before the formation of brood capsules and protoscoleces (Fig 1).

### Construction of cDNA libraries

Total RNA was extracted from Nonc (2 samples), Aonc, 4Wmet and Cmet using Trizol Reagent (Life Technologies). The mRNA was extracted using the Illumina mRNA Seq Sample Preparation Kit according to manufacturer instructions. Briefly, total RNA was subjected to poly (A) selection using Sera-Mag Magnetic Oligo-dT beads. Poly (A+) RNA was partially degraded by incubating in fragmentation buffer at 94°C for 5 min. The first-strand cDNA was synthesized using random primers and SuperScript II (Invitrogen), and the second-strand cDNA was synthesized using RNaseH and DNA pol I (Illumina). Illumina GA sequencing adaptors were ligated to the cDNA ends. Double-stranded cDNA was size-fractionated by 6% polyacrylamide gel electrophoresis (PAGE), and the band of 200 bp cDNA was recovered and amplified using Phusion DNA Polymerase (Finnzymes) in 15 cycles by PCR. Finally, 100 bp pair end read RNA-seq tags were generated using the Genome Analyzer IIx (Illumina, San Diego, CA, USA) following methods in the User Guide.

### RNA-Seq data analysis

RNA-Seq reads obtained from non-activated oncospheres, activated oncospheres and metacestodes were filtered by perl script using the following criteria: 1) trim adapter; 2) remove Illumina-filtered reads; 3) remove reads with no-call bases (ex: AATC "N" ATGATAG); and 4) remove mouse-mapped reads. RNA-seq reads were mapped to *E*. *multilocularis* genome version 3 (ftp://ftp.sanger.ac.uk/pub/project/pathogens/Echinococcus/multilocularis/genome/Emultilocularis_genome_v3.fas) using Illumina Eland (Elandv2), and the mapped read number for each gene was first transformed into reads per kilobase per million reads (RPKM), then filtered tRNA and rRNA coding genes. In addition, to validate the Next-Generation Sequencing (NGS) data, eight genes common to the Nonc1 and Cmet were selected for real-time PCR analysis. The primers employed for amplification of the eight genes and glyceraldehyde 3-phosphate dehydrogenase (EmuJ_000254600, internal control) were designed by OligoArchitect (http://www.oligoarchitect.com) and are shown in Table 1. The real-time PCR was performed using Applied Biosystems 7300 Real-time PCR System with SYBR-Green detection (SYBR Premix, TaKaRa) according to the manufacturer’s instructions. Each reaction was run in triplicate, after which the average threshold cycle (Ct) was calculated per sample and the relative expression of genes was calculated using the 2^-ΔΔCt^ method [27]. Different expressed genes were then identified by the edgeR package [28] with *p*<0.01 and false discovery rate (FDR) smaller than 0.05.

**Table 1:** Primers for real-time PCR.

Genes	Forward primers	Reserve primers
EmuJ_000212700	CGAAGGGTAATAAGGTGTA	TTGTAGAACTCACGATGT
EmuJ_000342600	CTTCATCCACATTATCACT	CAGTAGTAGCCAAGGATA
EmuJ_000355500	CGAAGGTGATGCTGAAGA	TCCGACCACAATGAAGAC
EmuJ_000372400	CGAAGTGCTCAAGTCTGA	GCTAGAGTCGGCATTGTA
EmuJ_000550000	AACTTCGTAGTCACTGAT	AGTCATCTCCTTGAACTT
EmuJ_001077100	TTCTTCTTCAATGCCATT	TACCTCCAGACTTGTTAG
EmuJ_001136900	TTCAATGCTACATCAGGTAAT	CGCCTACATTCCTTCTTAG
EmuJ_000770300	AACATGAGTGAGGAGAAT	CGTAGAACTTGTAGACATC
EmuJ_000254600	CTTCCAACTCTGTCAATG	GCTGTCAATAACCAACTT

### Antigen homologues in *E*. *multilocularis*

Putative antigen homologues of amino acid sequences in the *E*. *multilocularis* genome version 3 [23] were identified using known antigen sequences (accession numbers shown below). Briefly, BLASTP [29] comparisons were carried out using the amino acids sequences of *E*. *multilocularis* genome version 3 as queries and the known antigens sequences as subjects. Sequences with an E-value < 1E^-25^ and identity value > 80% were considered to be homologues of matched antigens within *Echinococcus* spp. Furthermore, antigen EG95 and diagnostic antigen gp50 family homologues were queried using the same amino acid sequences as used previously in the same genome version [23].

### Putative Em-TSP3 isoforms analysis

Integrative Genomics Viewer [30] was used to check the SNPs of the mapped reads at the putative Em-TSP3 isoform region of the scaffold. *De novo* assembled transcript sequences by the Trinity software [31] for each sample were compared with Em-TSP3 isoforms identified by BLASTX [15] using parameters–evalue 1e-20 -outfmt 6, and retained nucleotide sequences (S1 Table) showed more than 90% identity to isoforms of putative Em-TSP3. The putative amino sequences of retained nucleotide sequences were predicted by OrfPredictor [32], and aligned using Clustal Omega (http://www.ebi.ac.uk/Tools/msa/clustalo/).

### Accession numbers

Accession numbers for various known *E*. *multilocularis*, *E*. *granulosus* and *Taenia solium* antigens sequences used in this study are as follows: *E*. *multilocularis* (CAA59739, CAA10109, AAL51153, BAC11863, BAC66949, BAC77657, BAD89809, BAD89810, BAD89811, BAD89812, Q8WT41, BAF02516, BAF02517, BAF63674, BAF79609, ACJ02401, ACJ02402, ACJ02403, ACJ02404, ACJ02405, ACJ02406, ACJ02407, BAJ83490, BAJ83491, AER10547, AHA85399, Q07840, Q24895, Q24902, Q27652, Q8MM75, Q8WPI6, Q8WT42, Q9GP32, Q9NFZ5, Q9NFZ6, and Q9NFZ7); *E*. *granulosus* (AAF02297, AAL87239, CAF18421, AAX20156, AAX73175, ACA14465, ACA14466, ACA14467, ABI24154, AFI71096, AGE12481, AGE12482, O16127, O17486, O46119, P14088, P35417, P35432, Q02970, Q03341, Q03342, Q04820, Q07839, Q24789, Q24798, Q24799, Q24800, Q8MUA4, Q8T6C4, Q95PU1, Q9BMK3, Q9GP33, Q9GP38, Q9U408 and Q9U8G7); and *T*. *solium* (AAP49286, AAP49287, AAP49288, AAP49284 and AAP49285).

The raw sequence reads determined in this study have been deposited to DDBJ under the accession number DRA003058.

## Results and Discussion

### RNA-Seq data analysis

**Fig 2:**
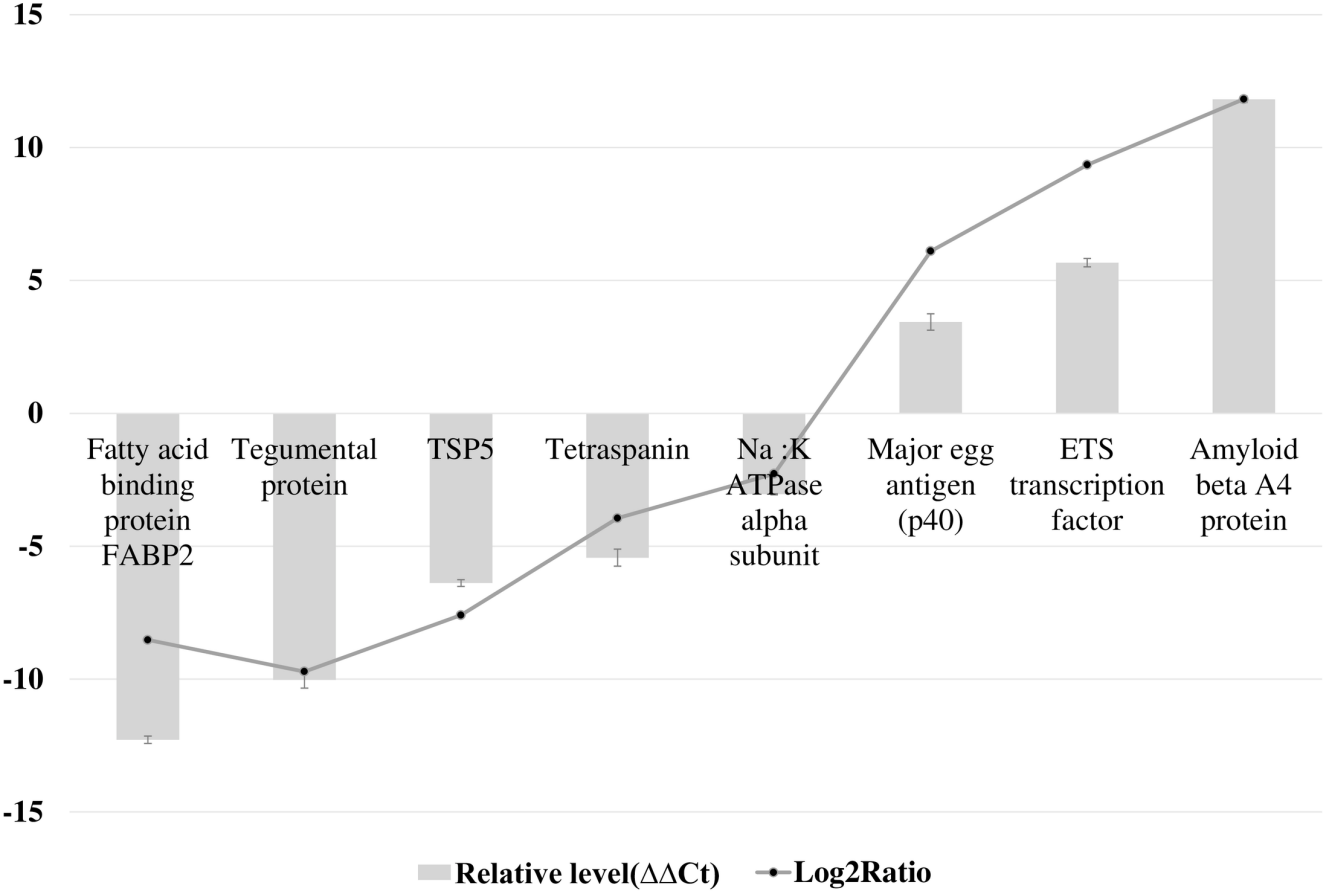
Real-time PCR validation of genes at kinds of expression level. The y-axis indicates the value of relative expression level (2^-ΔΔCt^) by real-time PCR and log_2_Ratio of Nonc1/Cmet by Next-generation sequencing. GAPDH as the internal control. Error Bar: Standard Deviation of the mean expression values of Real-time PCR.

We constructed five cDNA libraries from Nonc1, Nonc2, Aonc, 4Wmet and Cmet of *E*. *multilocularis* (Fig 1). More than 493 million clean reads were generated by Illumina paired-end sequencing, and 9,852 coding sequences of the genome were mapped with RPKM bigger than zero in at least one of the sequenced samples (S2 Table). The quality of obtained reads was excellent with more than 90% of reads having a quality score at Q30 (error probability of 0.001) or higher (Table 2). The results of real-time PCR analysis confirmed the NGS analysis data and show similar trends in fold change (Fig 2).

**Table 2:** Overview of the sequencing reads.

Samples	Yield (Mbases)	% PF*	Raw Reads	Clean Reads(mouse mapped reads filtered)	% of > = Q30 Bases (%PF)^#^	Mean Quality Score (%PF)
Nonc1	13,251	93.34	141,966,744	131,761,968	91.66	35.74
Nonc2	13,870	94.45	146,847,812	136,531,516	93.53	36.39
Aonc	12,735	94.76	134,400,788	126,184,658	93.48	36.24
4Wmet	12,430	93.77	132,558,666	231,874	91.36	35.79
Cmet	10,051	93.57	107,407,454	98,702,084	90.28	35.38

*%PF: The total fraction of passing filter reads assigned to an index.

^**#**^Q30:1 in 1000 base is mistake.

Nonc1: Non-activated oncosphere 1; Nonc2: Non-activated oncosphere2; Aonc: Activated oncosphere; 4Wmet: 4-week metacestodes miniature vesicles; Cmet: Metacestodes small vesicles cultivated *in vitro*.

**Fig 3:**
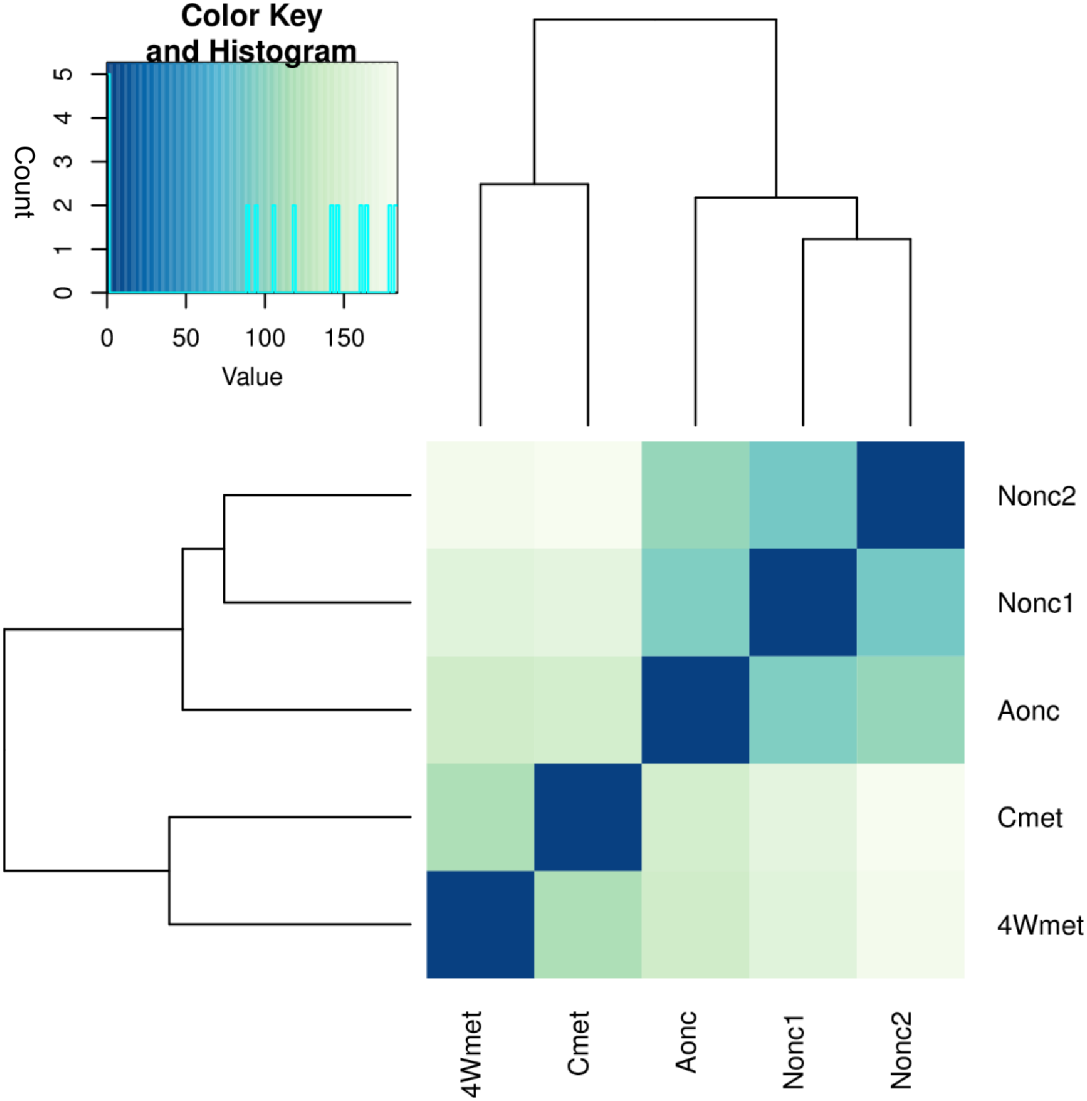
Heatmap showing the Euclidean distances between the samples as calculated from the variance stabilizing transformation of the count data by DEGseq pakage [33]. Nonc1: Non-activated oncosphere 1; Nonc2: Non-activated oncosphere2; Aonc: Activated oncosphere; 4Wmet: 4-week metacestodes miniature vesicles; Cmet: Metacestodes small vesicles cultivated *in vitro*.

**Fig 4:**
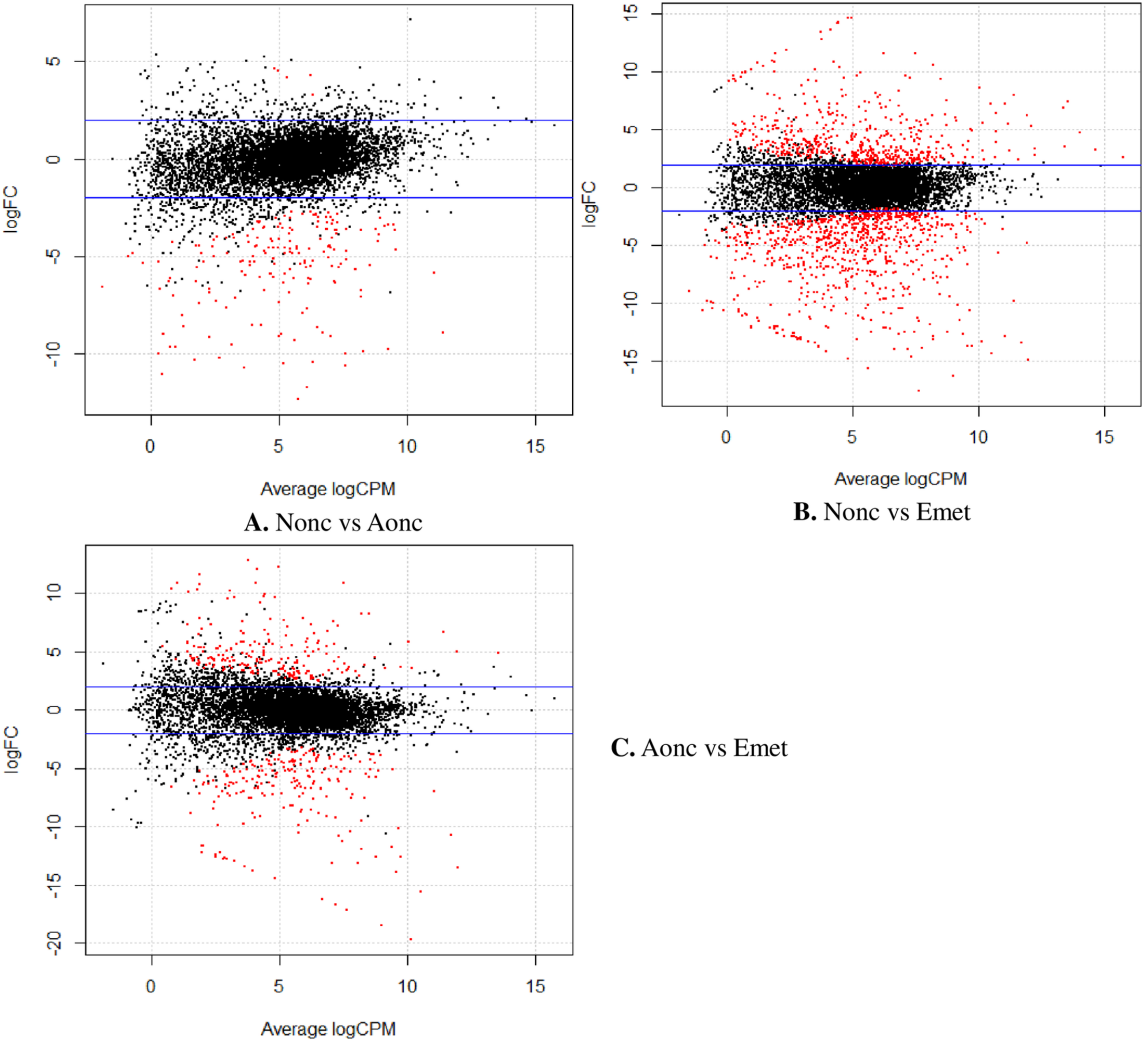
Analyses of differentially expressed genes (DEGs) among non-activated oncophere (Nonc), activated oncosphere (Aonc) and early stage of metacestode (Emet).

Larval tissue in the liver of 1–3 weeks post oral infections in DBA/2 mice were very small. After four weeks post oral infections, the lesions were identified in the livers and lesions with the parasite (4Wmet) were separated and extracted. The extracted sample contain more host tissue than the parasites which cause the number of reads were significantly decreased by filtering the mouse-mapped reads of 4Wmet (Table 2). But the cluster results showed a closer relationship with Cmet (Fig 3), which is in accordance with the biological development of *E*. *multilocularis*. For differentially expressed gene (DEG) analysis, we divided the cDNA libraries into biological development stages of non-activated oncosphere (Nonc1 and Nonc2), activated oncosphere (Aonc) and early stage metacestode (4Wmet, Cmet). There were 187 DEGs in the activated oncosphere versus non-activated oncosphere, 443 in activated oncosphere versus early stage metacestode and 1,433 in non-activated oncosphere versus early stage metacestode (Fig 4 and S2 Table). In total, there were 1,491 DEGs, and most of the genes identified between non-activated oncosphere versus early stage metacestode were also identified between activated oncosphere versus early stage metacestode (S2 Table). Almost DEGs were up-regulated when non-activated oncospheres transformed to activated oncospheres (Fig 4).

### Diagnostic antigen gp50 subunits in activated oncospheres

In this transcriptome datasets, 27 isoforms coding diagnostic antigen gp50 (GP50) mapped to 34 isoforms of the genome (S3 Table). Of the genome isoforms of GP50, 97.06% (33/34) were not expressed (RPKM<1) in Cmet, while 29.41% (10/34) were expressed (RPKM>1) in 4Wmet. Six GP50 isoforms were highly expressed (RPKM>100) in Aonc (Table 3), and five isoforms showed significantly higher expression (*p*<0.01, PDR<0.05) in the stage of activated oncospheres compared with non-activated oncospheres or the early stage metacestodes (Table 3). All six of the highly expressed GP50 had potential signal peptides, but only one had a GPI anchor (S3 Table).

**Table 3:** The antigen homologues matched to *E*. *multilocularis* proteome.

Gene ID	Accession number	Description	Species	Identity	E-value	Nonc1	Nonc2	Aonc	4Wmet	Cmet	Nonc vs Aonc	Nonc vs Emet	Aonc vs Emet
						RPKM	RPKM	RPKM	RPKM	RPKM	FDR	FDR	FDR
EmuJ_000364000	Q8MM75	14-3-3 protein homolog 2	*E*. *multilocularis*	99.58	8.00E-178	2,497.64	3,110.31	1,570.28	1,555.32	3,675.01	0.951	0.694	0.784
EmuJ_001192500	AAX73175	14-3-3 protein zeta	*E*. *granulosus*	98.39	0	1,737.74	2,488.74	2,087.08	2,202.17	1,785.08	1.000	0.635	0.979
EmuJ_000407200	P35432	ACTI	*E*. *granulosus*	97.87	0	0.28	0.22	16.30	9.20	250.48	0.006	0.000	0.258
EmuJ_000406900	P35432	ACTI	*E*. *granulosus*	97.07	0	0.26	0.42	30.92	4.60	433.97	0.023	0.000	0.458
EmuJ_000407300	Q03341	ACTII	*E*. *granulosus*	99.73	0	0.00	0.00	0.30	13.77	6.32	0.026	0.000	0.049
EmuJ_000006900	Q03341	ACTII	*E*. *granulosus*	88.1	2.00E-24	0.00	0.00	0.00	0.00	2.33	NA	NA	NA
EmuJ_000036300	Q03342	ACTIII	*E*. *granulosus*	98.38	0	2,990.42	895.40	1,196.83	668.68	785.70	1.000	0.166	0.932
EmuJ_000190400	Q03342	ACTIII	*E*. *granulosus*	98.38	0	2,877.54	1,631.00	1,204.67	799.28	709.87	0.921	0.071	0.939
EmuJ_000701700	Q03342	ACTIII	*E*. *granulosus*	94.57	0	0.04	0.03	0.15	941.02	78.61	1.000	0.000	0.000
EmuJ_000061200	Q03342	ACTIII	*E*. *granulosus*	94.5	0	0.76	0.63	1.64	3,007.91	219.08	1.000	0.000	0.000
EmuJ_000703300	Q03342	ACTIII	*E*. *granulosus*	94.5	0	0.01	0.00	0.21	653.62	21.65	0.285	0.000	0.001
EmuJ_000184900	AFI71096	Antigen 5	*E*. *granulosus*	96.69	0	71.79	22.04	18.02	157.05	750.33	0.922	0.008	0.017
EmuJ_001090800	Q07840	Antigen EM13	*E*. *multilocularis*	100	1E-54	711.79	77.84	569.71	87.38	99.98	1.000	0.095	0.183
EmuJ_000601200	AGE12481	Calcineurin A	*E*. *granulosus*	96.83	0	69.94	11.42	39.08	47.93	32.96	1.000	0.974	0.959
EmuJ_000447500	AGE12482	Calcineurin B	*E*. *granulosus*	98.79	3E-118	111.71	76.24	128.98	49.11	68.06	1.000	0.352	0.776
EmuJ_000454300	AER10547	Calcineurin B	*E*. *multilocularis*	100	4E-122	173.21	121.50	89.68	95.03	63.01	0.896	0.202	0.992
EmuJ_000920600	P14088	CYP-1	*E*. *granulosus*	99.38	5E-117	2,667.92	3,688.78	4,128.27	3,793.82	5,244.75	1.000	0.975	0.948
EmuJ_000517100	AAF02297	EF-1	*E*. *granulosus*	96.72	1E-177	1,247.18	1,550.50	1,026.26	664.44	516.83	1.000	0.119	0.907
EmuJ_000982200	BAF63674	EF1a	*E*. *multilocularis*	100	0	5,448.38	4,948.33	4,644.50	4,335.45	4,854.90	1.000	0.646	0.970
EmuJ_000342900	ABI24154	EG19	*E*. *granulosus*	84.93	2E-71	1.35	1.13	4.23	3.39	31.82	1.000	0.022	0.498
EmuJ_000485800	CAA10109	Elp (Antigen II/3)	*E*. *multilocularis*	100	0	3,971.70	3,429.81	3,767.91	1,741.57	692.90	1.000	0.063	0.567
EmuJ_000368620	AAL51153	EM95*	*E*. *multilocularis*	95.68	6E-111	17.36	39.73	6,843.39	237.19	0.10	0.055	0.690	0.078
EmuJ_000368610	AAL51153	EM95*	*E*. *multilocularis*	54.42	2.00E-41	0.00	0.00	0.00	0.00	0.00	NA	NA	NA
EmuJ_000375200	AAL51153	EM95*	*E*. *multilocularis*	31	4.00E-16	82.67	98.24	43.72	0.00	0.14	0.805	0.000	0.000
EmuJ_000328500	Q8WPI6	ONCO1*	*E*. *multilocularis*	99.36	7E-114	8,103.46	11,240.87	7,689.15	419.02	3.09	1.000	0.002	0.042
EmuJ_000710400	Q8WPI6	ONCO1*	*E*. *multilocularis*	41.67	4.00E-25	4,060.23	8,327.44	5,174.72	91.30	1.51	1.000	0.000	0.003
EmuJ_000381200	BAC77657	EmAgB8/1	*E*. *multilocularis*	100	1E-57	1.08	0.00	1.43	4,616.70	9,199.96	1.000	0.000	0.000
EmuJ_000381100	BAD89809	EmAgB8/2	*E*. *multilocularis*	100	8E-63	1.17	0.00	0.25	329.74	3,576.08	1.000	0.000	0.000
EmuJ_000381600	BAD89811	EmAgB8/3	*E*. *multilocularis*	100	1E-58	0.80	8.54	24.25	656.66	961.72	1.000	0.000	0.040
EmuJ_000381700	BAD89811	EmAgB8/3	*E*. *multilocularis*	100	1E-58	0.68	8.54	4.44	490.84	496.97	1.000	0.000	0.010
EmuJ_000381500	BAD89811	EmAgB8/3	*E*. *multilocularis*	92.05	6E-52	3.93	0.56	164.90	10,971.19	21,686.21	0.000	0.000	0.001
EmuJ_000381400	BAD89810	EmAgB8/4	*E*. *multilocularis*	100	2E-60	1.74	0.00	1.92	2,590.48	7,539.59	1.000	0.000	0.000
EmuJ_000381800	BAD89812	EmAgB8/5	*E*. *multilocularis*	100	4E-57	0.00	0.00	0.03	0.00	0.07	NA	NA	NA
EmuJ_000790200	BAJ83490	EmCBP1	*E*. *multilocularis*	100	0	2.48	1.42	114.41	232.80	445.61	0.000	0.000	0.387
EmuJ_000790300	BAJ83491	EmCBP2	*E*. *multilocularis*	100	0	7.57	2.49	40.14	20.42	19.16	0.201	0.137	0.855
EmuJ_000654500	BAF02516	EmCLP1	*E*. *multilocularis*	100	0	0.01	0.00	0.36	0.00	0.04	NA	NA	NA
EmuJ_000654600	BAF02516	EmCLP1	*E*. *multilocularis*	100	0	0.00	0.00	0.00	0.00	0.00	NA	NA	NA
EmuJ_000654100	BAF02516	EmCLP1	*E*. *multilocularis*	97.93	0	0.11	0.00	0.29	0.00	0.14	NA	NA	NA
EmuJ_000654200	BAF02516	EmCLP1	*E*. *multilocularis*	94.67	0	0.00	0.00	0.00	0.00	0.00	NA	NA	NA
EmuJ_000654800	BAF02516	EmCLP1	*E*. *multilocularis*	94.67	0	0.00	0.00	0.00	0.00	0.00	NA	NA	NA
EmuJ_000989200	BAF02517	EmCLP2	*E*. *multilocularis*	100	0	69.37	48.92	80.50	59.57	54.45	1.000	0.715	0.971
EmuJ_000941000	BAC66949	EmDLC	*E*. *multilocularis*	100	9E-56	3.77	3.10	98.82	163.54	721.24	0.004	0.000	0.252
EmuJ_000940900	BAC66949	EmDLC	*E*. *multilocularis*	88.31	7E-52	33.87	24.80	252.73	2,531.92	3,470.81	0.209	0.000	0.025
EmuJ_000590100	BAC66949	EmDLC	*E*. *multilocularis*	83.12	4E-46	36.66	34.27	64.78	100.36	123.22	1.000	0.354	0.749
EmuJ_000941100	BAC66949	EmDLC	*E*. *multilocularis*	83.12	1E-49	5.12	6.98	64.69	124.08	946.12	0.379	0.000	0.147
EmuJ_000946700	BAC66949	EmDLC	*E*. *multilocularis*	83.12	2E-48	324.29	647.14	812.70	25.62	7.96	1.000	0.000	0.000
EmuJ_001060400	BAC66949	EmDLC	*E*. *multilocularis*	83.12	8E-46	127.77	180.55	268.92	474.48	763.07	1.000	0.212	0.561
EmuJ_000940800	BAC66949	EmDLC	*E*. *multilocularis*	81.82	1E-48	0.96	0.57	15.94	37.20	120.86	0.061	0.000	0.331
EmuJ_000538300	CAA59739	EMGST1	*E*. *multilocularis*	100	1E-165	553.09	531.84	697.37	1,395.50	1,515.60	1.000	0.474	0.642
EmuJ_000791700	BAC11863	EmTRX	*E*. *multilocularis*	100	2E-146	1,182.52	1,611.54	2,307.76	2,834.87	2,684.39	1.000	0.808	0.920
EmuJ_000515900	BAF79609	EMY162	*E*. *multilocularis*	98.04	1E-109	0.08	0.10	3.98	0.00	19.00	0.133	0.002	0.775
EmuJ_000021700	BAF79609	EMY162	*E*. *multilocularis*	89.32	1E-67	0.00	0.00	0.03	0.00	0.00	NA	NA	NA
EmuJ_000564900	BAF79609	EMY162	*E*. *multilocularis*	84.67	4E-92	48.88	162.47	639.91	73.97	146.34	0.935	0.677	0.258
EmuJ_000492700	AAL87239	EpC1	*E*. *granulosus*	94.37	4E-43	10.88	7.44	75.29	954.51	1,717.34	0.256	0.000	0.012
EmuJ_000550000	Q02970	FABP1	*E*. *granulosus*	93.23	9E-90	1.61	2.16	61.61	361.74	593.83	0.001	0.000	0.052
EmuJ_000549800	Q9BMK3	FABP2	*E*. *granulosus*	99.25	1E-95	0.14	0.37	1.66	167.93	199.22	0.899	0.000	0.000
EmuJ_000905600	Q9GP32	FBPA	*E*. *multilocularis*	99.72	0	946.48	570.08	349.68	1,013.07	3,354.39	0.921	0.363	0.161
EmuJ_000382200	O46119	Ferritin	*E*. *granulosus*	96.53	2E-125	5,317.20	7,184.38	4,466.66	5,359.65	14,599.80	1.000	0.944	0.669
EmuJ_000254600	Q27652	GAPDH	*E*. *multilocularis*	96.9	0	7,190.54	952.35	2,507.29	3,511.48	9,918.31	1.000	0.618	0.585
EmuJ_000515500	AAP49287	GP50b*	*T*. *solium*	40.75	1.00E-67	442.74	503.43	174.93	4.24	0.12	0.770	0.000	0.000
EmuJ_000261100	AAP49287	GP50b*	*T*. *solium*	40.87	5.00E-65	0.06	0.58	1,203.70	56.40	0.21	0.001	0.030	0.087
EmuJ_000049700	AAP49287	GP50b*	*T*. *solium*	34.47	5.00E-43	32.51	13.58	542.58	184.22	0.01	0.543	0.584	0.633
EmuJ_000512300	AAP49287	GP50b*	*T*. *solium*	33.05	7.00E-43	0.00	0.11	499.58	14.91	0.00	0.000	0.024	0.029
EmuJ_000293700	AAP49288	GP50c*	*T*. *solium*	37.58	9.00E-68	431.40	537.43	420.06	5.96	0.02	1.000	0.000	0.000
EmuJ_000681200	AAP49288	GP50c*	*T*. *solium*	33.22	2.00E-43	8.85	4.21	279.76	334.10	6.83	0.019	0.006	0.928
EmuJ_000249600	Q24895	GRP78(HSP70)	*E*. *multilocularis*	99.85	0	185.09	159.39	372.98	266.34	222.14	1.000	0.987	0.953
EmuJ_000212700	Q8WT42	HSP20(Onco2)	*E*. *multilocularis*	100	0	6,878.11	6,212.35	1,352.09	388.39	100.30	0.330	0.000	0.205
EmuJ_001085100	Q24789	HSP70	*E*. *granulosus*	96.54	0	225.68	28.47	838.10	377.73	359.20	0.271	0.212	0.783
EmuJ_000723700	Q24789	HSP70	*E*. *granulosus*	91.73	5E-152	7.80	0.82	13.62	6.81	14.43	1.000	0.396	1.000
EmuJ_001085400	Q24789	HSP70	*E*. *granulosus*	90.73	0	3,095.23	1,216.16	2,699.23	1,063.90	1,557.45	1.000	0.472	0.838
EmuJ_000417100	Q04820	MDH	*E*. *granulosus*	97.89	0	192.94	195.02	514.29	925.42	2,753.59	1.000	0.018	0.396
EmuJ_001185000	CAF18421	Mdhm	*E*. *granulosus*	99.11	0	28.21	26.27	96.18	95.32	105.92	1.000	0.171	0.945
EmuJ_001185100	CAF18421	Mdhm	*E*. *granulosus*	99.04	0	35.39	48.46	275.68	194.92	190.08	0.614	0.171	0.971
EmuJ_000653900	Q24799	Myophilin	*E*. *granulosus*	98.82	5E-125	5.44	12.99	4.49	18.76	316.68	1.000	0.020	0.048
EmuJ_000550800	AHA85399	P29	*E*. *multilocularis*	99.58	6E-177	326.60	307.02	375.86	314.76	521.18	1.000	0.978	0.932
EmuJ_000763300	P35417	Paramyosin	*E*. *granulosus*	98.96	0	0.21	0.16	0.25	19.36	200.49	1.000	0.000	0.000
EmuJ_001193100	Q8WT41	Serpin^Emu^	*E*. *multilocularis*	100	0	197.45	206.12	27.81	19.50	9.69	0.068	0.000	0.860
EmuJ_001193200	Q8WT41	Serpin^Emu^	*E*. *multilocularis*	99.72	0	158.01	165.38	23.16	6.49	1.66	0.066	0.000	0.160
EmuJ_000372400	AAX20156	Tegumental protein	*E*. *granulosus*	84.31	4E-25	8.96	11.48	9,293.21	20,701.84	7,556.48	0.000	0.000	0.861
EmuJ_000602100	O17486	Thioredoxin	*E*. *granulosus*	98.13	7E-67	89.06	101.37	140.04	99.31	118.35	1.000	0.834	0.993
EmuJ_000958100	Q95PU1	Tropomyosin	*E*. *granulosus*	100	0	147.90	59.78	142.03	89.08	63.22	1.000	0.532	0.844
EmuJ_000355800	ACJ02402	Em-TSP1	*E*. *multilocularis*	100	0	0.12	0.16	8.06	139.89	111.06	0.000	0.000	0.010
EmuJ_001070300	ACJ02403	Em-TSP2	*E*. *multilocularis*	100	2E-148	24.46	23.81	69.16	175.63	176.58	1.000	0.012	0.429
EmuJ_000328400	ACJ02404	Em-TSP3	*E*. *multilocularis*	100	6E-107	19.95	0.08	6.47	0.00	0.45	1.000	0.062	0.278
EmuJ_001077400	ACJ02404	Em-TSP3	*E*. *multilocularis*	100	6E-107	229.08	0.07	76.69	40.67	1.38	1.000	0.412	0.777
EmuJ_000289200	ACJ02404	Em-TSP3	*E*. *multilocularis*	91.22	5E-97	0.00	0.00	0.00	0.00	0.00	NA	NA	NA
EmuJ_001077200	ACJ02404	Em-TSP3	*E*. *multilocularis*	91.22	5E-97	0.00	0.00	0.00	0.00	0.00	NA	NA	NA
EmuJ_001077300	ACJ02404	Em-TSP3	*E*. *multilocularis*	91.22	5E-97	229.06	0.07	76.69	40.67	1.13	1.000	0.416	0.777
EmuJ_001021500	ACJ02405	Em-TSP4	*E*. *multilocularis*	100	9E-167	14.97	18.48	73.14	415.80	152.32	0.952	0.001	0.277
EmuJ_001077100	ACJ02406	Em-TSP5	*E*. *multilocularis*	100	1E-161	15.32	14.63	668.99	1,062.25	2,961.54	0.001	0.000	0.471
EmuJ_001021300	ACJ02407	Em-TSP6	*E*. *multilocularis*	99.55	1E-161	62.14	83.07	78.16	353.52	310.79	1.000	0.152	0.219
EmuJ_000834300	ACJ02401	Em-TSP7	*E*. *multilocularis*	100	2E-168	59.30	38.08	86.46	72.75	127.43	1.000	0.559	0.893
EmuJ_000202600	Q9NFZ7	TUB-1	*E*. *multilocularis*	100	0	0.80	1.31	13.77	364.22	578.46	0.158	0.000	0.006
EmuJ_000672200	Q9NFZ6	TUB-2	*E*. *multilocularis*	100	0	745.95	531.67	1,037.80	1,444.02	2,979.22	1.000	0.249	0.637
EmuJ_000069900	Q9NFZ6	TUB-2	*E*. *multilocularis*	96.51	0	0.63	0.08	0.53	102.21	0.26	NA	NA	NA
EmuJ_000569000	Q9NFZ6	TUB-2	*E*. *multilocularis*	96.28	0	0.19	0.05	1.46	5.18	1.90	0.286	0.004	0.784
EmuJ_000955100	Q9NFZ6	TUB-2	*E*. *multilocularis*	96.05	0	0.48	0.39	1.01	2.59	0.51	1.000	0.681	0.975
EmuJ_000617000	Q9NFZ6	TUB-2	*E*. *multilocularis*	94.19	0	0.02	0.00	0.04	406.27	0.23	NA	NA	NA
EmuJ_000041100	Q9NFZ6	TUB-2	*E*. *multilocularis*	80.14	0	1.91	0.62	5.07	6.47	1.09	1.000	0.578	0.965
EmuJ_000202500	Q9NFZ5	TUB-3	*E*. *multilocularis*	100	0	23.71	12.67	296.90	647.27	1,307.92	0.017	0.000	0.355

*Identified by previous study [23]

Nonc: Non-actvated oncospheres; Aonc: Activated oncospheres; Emet: Early stage metacestodes.

NA: Genes were filtered when executed significant gene expression.

**Fig 5:**
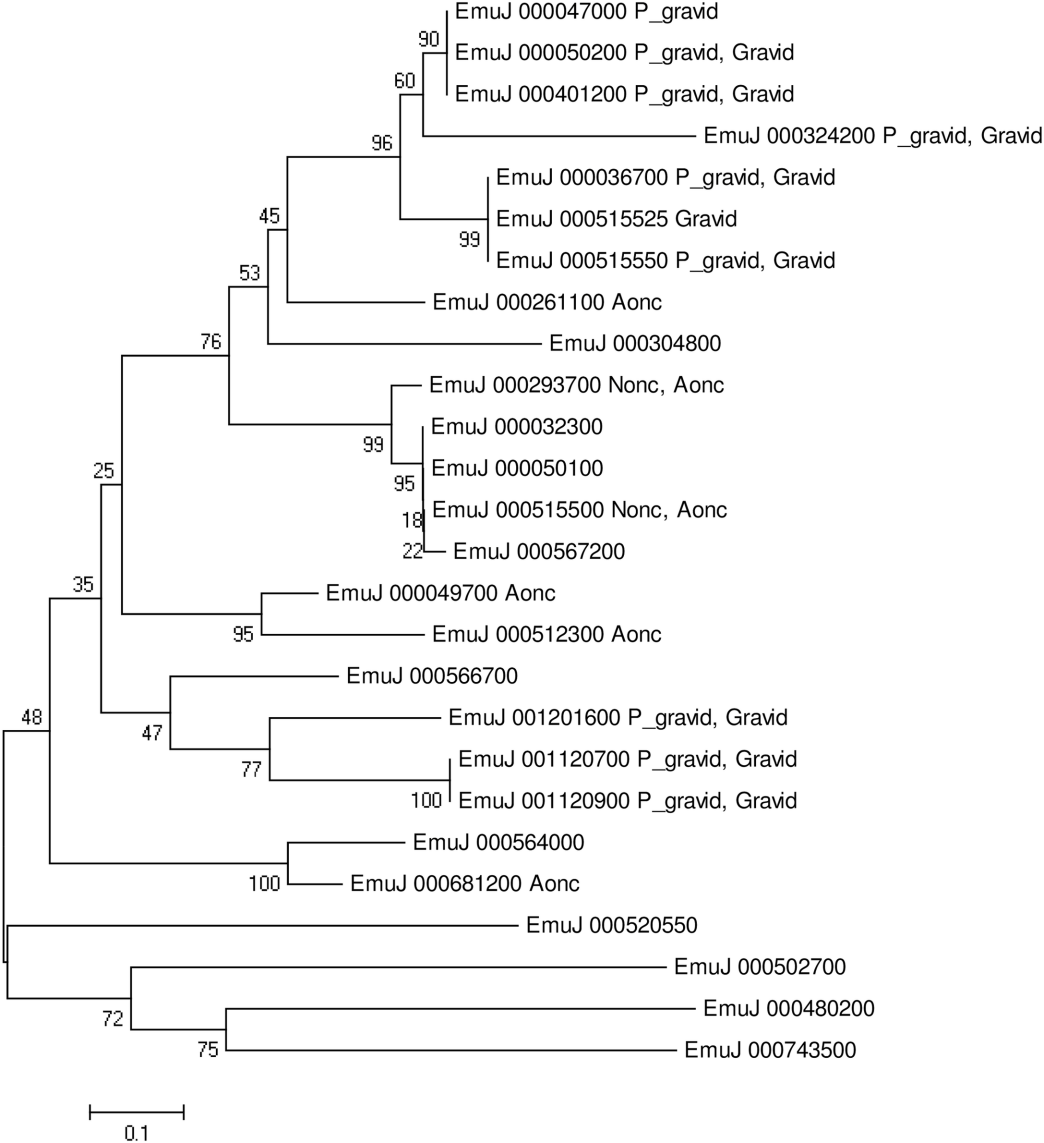
Phylogenetic analyses of diagnostic antigen gp50 amino acid sequences. The neighbor-joining tree was constructed by MEGA 6.0 (www.megasoftware.net); bootstrap values were obtained from 1,000 replicates. Nonc: RPKM>100 at stages of non-activated oncosphere; Aonc: RPKM>100 at stages of activated oncosphere; P_gravid: FPKM>100 at stage of pre-gravid [23]; Gravid: FPKM>100 at stage of gravid [23].

*Taenia solium* GP50 has been used for the diagnosis of cysticercosis [34]. GP50 isoforms are species-specific antigens and may be stage-specific in *Cysticercus cellulosae* [35] based on the lack of antibody reactivity with one serum sample from an individual confirmed to be taeniasis-positive but cysticercosis-negative [35]. A previous study showed that more than 90% of *E*. *multilocularis* GP50 isoforms were not expressed in metacestodes cultivated *in vitro* [23], and our present work also corroborated this finding, as few or no transcripts of GP50 were found in Cmet. Some GP50 isoforms were expressed in 4Wmet from *in vivo* DBA/2 mice infections, suggesting that these GP50 isoforms are key factors in the host-parasite interface during the early stage of infection. GP50 antigen family expression also showed quite high variability (Fig 5), and the lack of uniformity of isoform expression in oncospheres (non-activated and activated) and adults (pre-gravid and gravid) (Fig 5) indicates that the *E*. *multilocularis* diagnostic antigen GP50 may be stage-specific as well.

### EG95 (Fibronectin type III-like) in activated and non-activated oncospheres

Previous studies have described the effectiveness of Fibronectin type III domain-like protein vaccines against echinococcosis [15, 36, 37]. These highly immunogenic proteins, which may be involved in host invasion, are encoded by a multigene family; EG95 vaccine is effective against *E*. *granulosus*, and EM95 is effective against *E*. *multilocularis* [36, 38]. The antigen is a secreted protein with a GPI anchor that is upregulated during oncosphere activation [38, 39] and is probably involved in cell adhesion [40]. Three (EmuJ_000328500, EmuJ_000368620, EmuJ_000710400) out of five EG95 relatives followed the previous prediction [23], and corresponded to the top 20 expressed proteins in non-activated and activated oncospheres (S2 Table). Unlike EmuJ_000328500 and EmuJ_000368620, the highly expressed EmuJ_000710400 showed low identity with the published EM95 antigens (Table 3), suggesting that it may be a new candidate antigen for vaccine development against alveolar echinococcosis. Most interestingly, EmuJ_000368620 which shows highest identity to EM95 is significantly expressed in activated oncospheres (Table 3). However, EmuJ_000328500 which shows highest identity to ONCO1 (79.5% identity to EM95) is highest expression in non-activated oncospheres (Table 3). It is not surprised that EmuJ_000328500 has the highest expression level in the non-activated oncospheres in accordance with the data from previous study [41].

EMY162, a potential vaccine candidate against *E*. *multilocularis*, showed 31.4% identity to the amino acid sequence of EM95, which is also a fibronectin type III-containing protein [15].

EmuJ_000564900 (85% identity to BAF79609) was expressed in most of the life-cycles stages, especially in activated oncospheres (Table 3), EmuJ_000021700 (89% identity to the BAF79609) showed almost no expression in sequenced samples of our present work (Table 3), and EmuJ_000515900 (98% identity to the BAF79609) primarily expressed in cultured small vesicles in our study (Table 3), which is consistent with findings in a previous study [15, 23].

### Serine protease inhibitors predominated in non-activated oncosphere

Serpins (serine proteinase inhibitors) constitute a huge family of about 1,500 identified members. The function of serpins ranges from the regulation of proteinases from immune effector cells, blood coagulation and in the complement system in mammals [42]. The serpin of *E*. *multilocularis* (serpin^Emu^) was the first member described from this class of cestodes [41], and sequence analysis indicated that it was an intracellular serpin [41, 43]. The putative amino acid sequences of the parasite genome data [23] suggested that serpin^Emu^ with a signal peptide predicted by Phobius [44]. In addition, *in vitro* assays have confirmed that serpin^Emu^ fails to inhibit cathepsin G and chymotrypsin but can readily inhibit trypsin and pancreatic elastase [43], both of which are digestive enzymes in the intestines of mammals. Therefore, an extracellular role of serpin^Emu^ may be possible. Previous descriptions of the ultrastructure of *E*. *granulosus* oncospheres have referred to the penetration gland cells [45] and proteinases may make up a considerable portion of the excreted proteins during the penetration process that is hypothesized to involve the secretion that may help the parasite penetrate the intestinal wall of the intermediate host [6, 7,45, 46]. If serpin^Emu^ is excreted by penetration gland during the infection phase of the oncospheres, it might be able to block the proteolytic attack of host digestive enzymes. If so, it may even be a target of the intestinal immune system and a vaccine candidate.

### HSPs antigens constantly expressed in sampled life-cycle stages

The putative HSP20 gene, which can express immunogenic products and stimulate the immune system, showed high expression in the oncosphere stage [41, 47]. The predicted HSP20 homologue (onco2) also showed the highest expression at the stages of non-activated oncosphere (RPKM = 6, 545.23) and also showed expression at the activated oncosphere stage and in early stage metacestodes as well (Table 3). Taken together with the findings from the published transcriptome of *E*. *multilocularis* [23], it is clear that this molecule was expressed at almost all stages of *E*. *multilocularis*, including non-activated oncosphere, activated oncosphere, metacestode and adult worms.

The HSP70 family, which has been described as the major antigens in *Echinococcus* spp.[48, 49] and are the most striking gene family expansions with 22 full copies in *E*. *multilocularis* genomes version 3 [23]. Furthermore, in various infectious disease models including echinococcosis, vaccination strategies using HSPs have produced significant protection [48, 50]. The transcriptome datasets of the present study show that HSP70 homologues were constantly expressed in all stages (Table 3). Continuous antigenic stimulation with parasite-derived HSP families would induce an apparent antibody response to these molecules in infected animals. These antibody responses create an opportunity to use HSPs in diagnostic assay and vaccine development for echinococcosis.

### Antigen II/3 homologues constantly expressed in sampled life-cycle stages

Antigen II/3 share homology with the mammalian ezrin/radixin/moesin (ERM) protein family that is involved in several key processes related to cellular architecture, including cell-cell adhesion, membrane trafficking, microvillus formation and cell division [51]. Antigen II/3 is encoded by the elp gene and the antigens of Em10 and Em18 are thought to be homologues, which have also been used as important diagnostic antigens [52, 53]. In the present study, antigen II/3 was highly expressed in all sequenced samples, but it had a relative higher expression level in non-activated and activated oncospheres. Previous studies proved that antigen II/3 can be expressed at the stages of protoscoleces, metecestode and adult and are localized within the germinal layer and parenchymal cell of protoscoleces and on the surface of calcareous corpuscles [52]. Even though expression is relatively low in Cmet, there was no significant difference compared with the other collected data (Table 3). It has been shown that antigen II/3 is also constantly expressed in the early stage metacestodes and adults (FPKM>200 [23]).

The viability of protoscoleces was significantly reduced at day 10 after silencing the elp gene statistically [54]. Together with the constantly high expression level of antigen II/3 at almost all life-cycle stages may hint that antigen II/3 has a fundamental role for supporting parasites, such that antigen II/3 can act not only as an important diagnostic antigen special for the oncosphere stage, but also as a vaccine candidate.

### AgB subunit expression in non-activated and activated oncospheres

Antigen B (AgB) was initially identified as major hydatid cyst fluid antigen of *E*. *granulosus* [55]. In *E*. *multilocularis* genome version 3, there are seven isoforms that code antigen B subunits, of which EmAgB8/3 (EmuJ_000381500) had the highest expression (RPKM = 21, 686.21) among known antigens (Table 3) and was third highest expression of transcriptome of Cmet (S2 Table); even activated oncospheres showed relative high expression (RPKM = 164.90). In addition, the three isoforms that code EmAgB3 were expressed not only in the early stage metacestode but also in the adult [23] and non-activated and activated oncospheres (Table 3). Unlike other AgB subunits, which were almost within the 2-fold expression level of 4Wmet and Cmet, EmAgB2 showed a more than 10-fold difference. Previous studies have shown that the sensitivity of EgAgB2 was obviously different in different assays [14, 56], and one reason may be that *E*. *granulosus* isolated from CE patients in different countries expresses differing levels of the AgB2 subunit [56]. Our data suggest this might be caused by differing expression of AgB2 within the early stage metacestodes. Furthermore, antibody responses to AgB in different cyst stages of different sensitivities [4] also indicate that AgB subunits dynamically change in cyst stages. In conclusion, from the perspective of expression level, we proposed that EmAgB8/3 may be expected to have essential metabolic functions throughout all life-cycle stages of the parasite, while EmAgB8/1, EmAgB8/2, and EmAgB8/4 may be essential factors for survival of larvae in intermediate hosts. EmAgB8/5, which was firstly detected to be highly expressed in the adult of *E*. *multilocularis* [57], but was not detected in this study.

### Some Em-TSPs homologues with stages-specific expression

**Fig 6:**
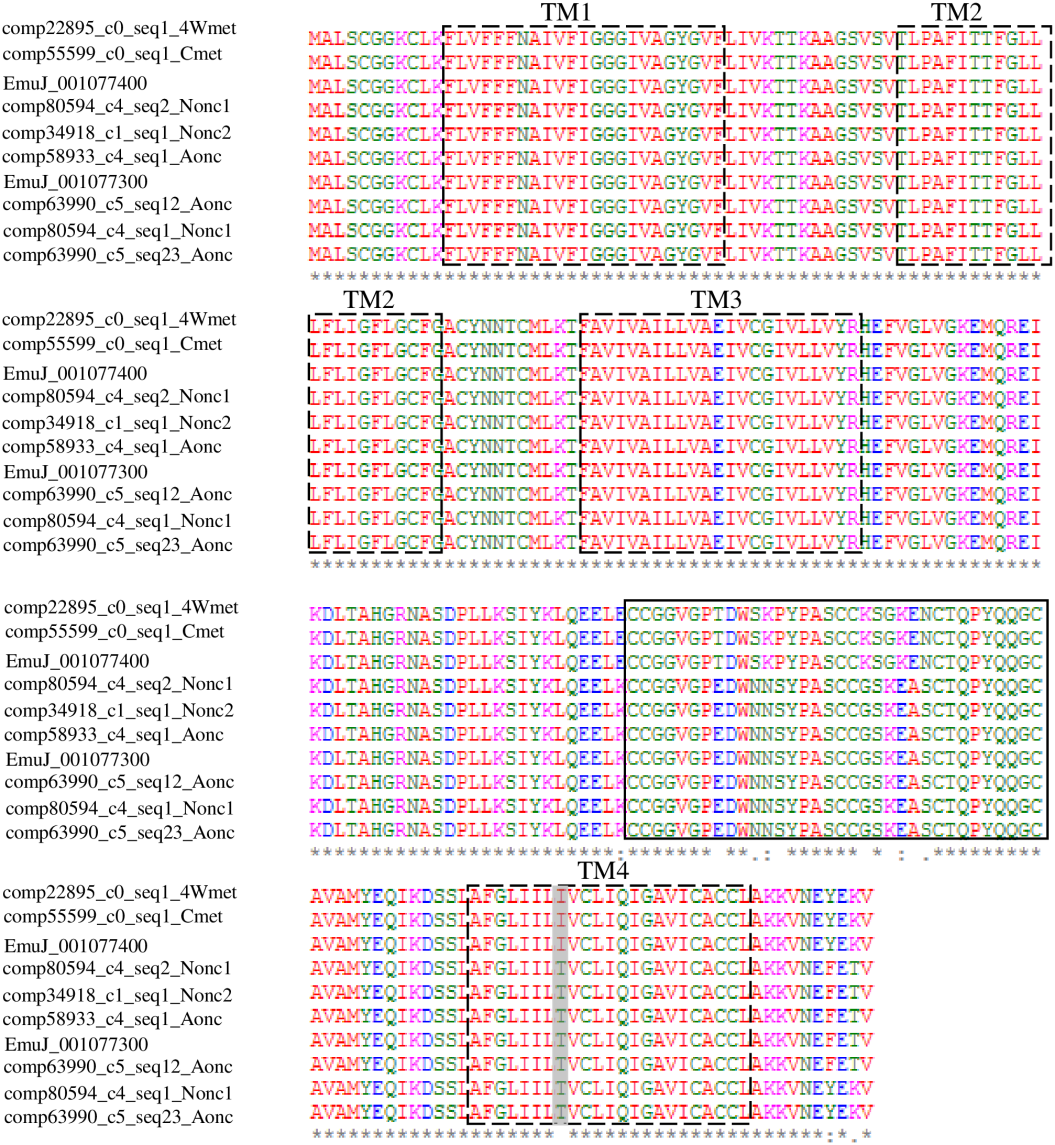
Protein alignment of putative Em-TSP3 isoforms with four transmembrane. Fully conserved residues are marked with (*), those replaced with amino acids of strongly similar properties with (:) and of weakly similar properties with (.) LEL variable region are in the solid line box and predicted transmembrane region are in the dashed line box. Protein mutant of the forth transmembrane is shaded in grey. There are three Em-TSP3 isoforms predicted, two of them are conserved with previous study [23], but one of them are intermediate type of the former two isoforms and need further verification.

Tetraspanins (TSPs) are a superfamily of plasma membrane-associated proteins consisting of four conserved transmembranes [58]. They have been used as vaccine candidates against schistosomiasis, echinococcosis and as diagnostic antigens for cysticercosis [2, 20, 59, 60]; In addition, it was proven that tetraspanins in the tegument of schistosomula and adult worms can act as receptors for host ligands, including MHC molecules, allowing parasites to mask their non-self-status and escape host immune responses [61]. A total of 11 amino acid sequences (Table 3) showed 91%-100% identity to the seven published Em-TSPs [20]. In addition, there were two putative Em-TSP3 isoforms and two amino acid sequences of one isoform and three amino sequences of another isoform (Table 3), and most mutation sites were located at the LEL variable region (Fig 6).

Previous transcriptome data [23] and the present study showed that Em-TSP5 is expressed at almost all life-cycle stages and is significantly expressed at the stage of activated oncospheres and early stage metacestodes compared with non-activated oncosphere (Table 3). Em-TSP5 was intensely stained in sections of the germinal layer of metacestode [20]. Em-TSP5 is closely related to the T24 antigen of *T*. *solium*, a diagnostic antigen for cysticercosis [60], which suggest that Em-TSP5 may be an important diagnostic candidate for detecting early stage infection.

Em-TSP1, one of the highly protective vaccine candidates [20], is located at the surface (germinal layer/tegument) of *E*. *multilocularis* larvae and the tegument of the adult worms. Significantly high expression in early stage metacestode compared with non-activated and activated oncospheres was observed (Table 3). A previous study showed that another protective effect vaccine candidate, Em-TSP3, is localized in the non-activated oncospheres and protoscoleces and the germinal layer of *E*. *multilocularis* cysts [2]; the genome-mapped data in the present study showed relative higher expression in Aonc and 4Wmet than in Cmet (no protoscoleces), and the expression level of Em-TSP3 varied within non-activated oncospheres samples (Table 3). However, the *de novo* assembled data (S4 Table) showed that Em-TSP3 homologues were highly expressed in both samples of non-activated oncospheres. In addition, the RPKM data in the present study could not distinguish the expression difference between two putative Em-TSP3 isoforms located in the same scaffold (pathogen_EMU_scaffold_007780, EmuJ_001077300 (pEm-TSP3-1), EmuJ_001077400 (pEm-TSP3-2)) (Table 3) of the parasite genome, and the visualization mapped reads showed that almost all reads from different samples can be simultaneously mapped to EmuJ_001077300 and EmuJ_001077400 genes, but there was an obvious SNP (G/A) of the mapped reads at the mapped positions of 13002639 and 13008462 in the scaffold and also in the fourth transmembrane region of the putative amino acid sequences of EmuJ_001077300 and EmuJ_001077400 that cause threonine to change to isoleucine (Fig 6 and S1 Fig). Namely, 97% (8911, 8669G and 217,183A), 90% (1428, 1398G and 147, 146A), 78% (7,7G and 2, 2A) and 34% (11,11G and 21, 21A) of mapped reads were guanine of Nonc1, Aonc, 4Wmet and Cmet, respectively. The sequences from the *de novo* assembled data that showed 100% identity to EmuJ_001077300 were highly expressed and those with 100% identity to EmuJ_001077400 had relatively lower expression for Nonc and Aonc (S4 Table). Furthermore, no transcripts showed 100% identity to EmuJ_001077300 in Cmet, and the expression level that showed 100% identity to EmuJ_001077400 was similar to oncosphere (S4 Table). Together with mapped data and *de novo* assembled data, it is considerable that EmuJ_001077400 may be constantly expressed in oncospheres and metacestodes at a normal level and EmuJ_001077300 may show specific high expression in the oncospheres. The relatively higher ratio of guanine at polymorphic site in 4Wmet was also found to be different from that in Cmet but similar to those in oncospheres. Interestingly, the *de novo* assembled data identified an intermediate type isoform of pEm-TSP3 at the oncosphere stage (Fig 6), which needs further verification.

Growing evidence suggests the importance of Th1/Th2 balance during parasite infections. Previous studies has shown that rEm-TSP3 may cause Th1 and Th2 responses by different immunization routes with Th2 being predominant [2]. A recent study [62] showed that rEg-TSP1 (95% identity to Em-TSP1) may cause a Th1 response. The stage-specific expression of Em-TSPs, especially Em-TSP1 and Em-TSP3, which are two of the most effective vaccines against echinococcosis, showed almost opposite expression at the same stage, suggesting that the two Em-TSPs may influence each other. Together with the ‘tetraspanin web’, which can lead to the dynamic assembly of tetraspanin family proteins dependent on the ability of its members to form lateral associations with multiple partner proteins and with each other [63]. We propose that Em-TSP1 and Em-TSP2 can down-regulate each other. Further confirmation of the mutual inhibition of these two Em-TSPs may require a challenge experiment conducted *in vivo* or *in vitro*. The fact that some tetraspanin proteins cross-react with several others implies that immunization with one tetraspanin antigen could block several tetraspanins functions [20] and the highly expressed of pEm-TSP3-1 in oncospheres hint that it could act as a specific vaccine in the early phase of infection.

### Conclusion

In this study, we have conducted RNA-Seq analysis of the oncospheres and early stage metacestodes of *E*. *multilocularis* (Nemuro strain). A global view of gene expression profiles and the stage-specific significant different express genes are revealed during the early invasion phases of the parasite. Further analysis show that tapeworm-specific AgB antigen family dominated in early stage metacestodes, GP50 antigen family dominated in activated oncospheres and Eg95 antigen are dominated in non-activated and activated oncospheres. In addition, heat shock proteins and antigen II/3 which contain highly conserved domain in invertebrates and vertebrates are constantly expressed in the three stages. The reveal of various known antigens expression level during the parasite development stages, especially the stages of non-activated and activated oncospheres, will give fundamental information for choosing candidate genes used in early diagnosis.

## Supporting Information

S1 Fig IGV view of putative Em-TSP3 isoforms in pathogen_EMU_scaffold_007780 of *E*. *multilocularis* genome versions 3. The five tracks show the mapped result of EmuJ_001077300 (gene start: 13002559, gene end: 13003475) and EmuJ_001077400 (gene start: 13008382, gene end: 13009298) which all most reads can mapped to the two putative Em-TSP3 isoforms. At the locus of 13002639 and 13008462, there was two common single-nucleotide polymorphisms (SNPs) but the ratio of the two SNPs were obviously different among the samples.
(PPTX)

S1 Table Nucleotide sequences of putative Em-TSP3. (XLSX)

S2 Table Significant different expressed genes in Non-activated oncospheres, Activted oncospheres and early stage of Metacestodes from Echinococcus multilocularis (P<0.01, FDR<0.05). (XLSX)

S3 Table Transcript data of dignostic antigen gp50 family in any of the sampled life cycle stage in this study and previous report [23]. (XLSX)

S4 Table BLASTX results of putative Em-TSP3 isoforms from different samples. (XLSX)
