# Supplementary material for: Analysis on Gene Expression Profile in Oncospheres and Early Stage Metacestodes from Echinococcus multilocularis
Source: PLoS Negl Trop Dis. 2016 Apr 19;10(4):e0004634. doi: 10.1371/journal.pntd.0004634 (PMC4836691; doi:10.1371/journal.pntd.0004634)
Supplement: S1 Fig — The five tracks show the mapped result of EmuJ_001077300 (gene start: 13002559, gene end: 13003475) and EmuJ_001077400 (gene start: 13008382, gene end: 13009298) which all most reads can mapped to the two putative Em-TSP3 isoforms. At the locus of 13002639 and 13008462, there was two common single-nucleotide polymorphisms (SNPs) but the ratio of the two SNPs were obviously different among the samples. (PPTX) [file pntd.0004634.s001.pptx]

## Slide 1
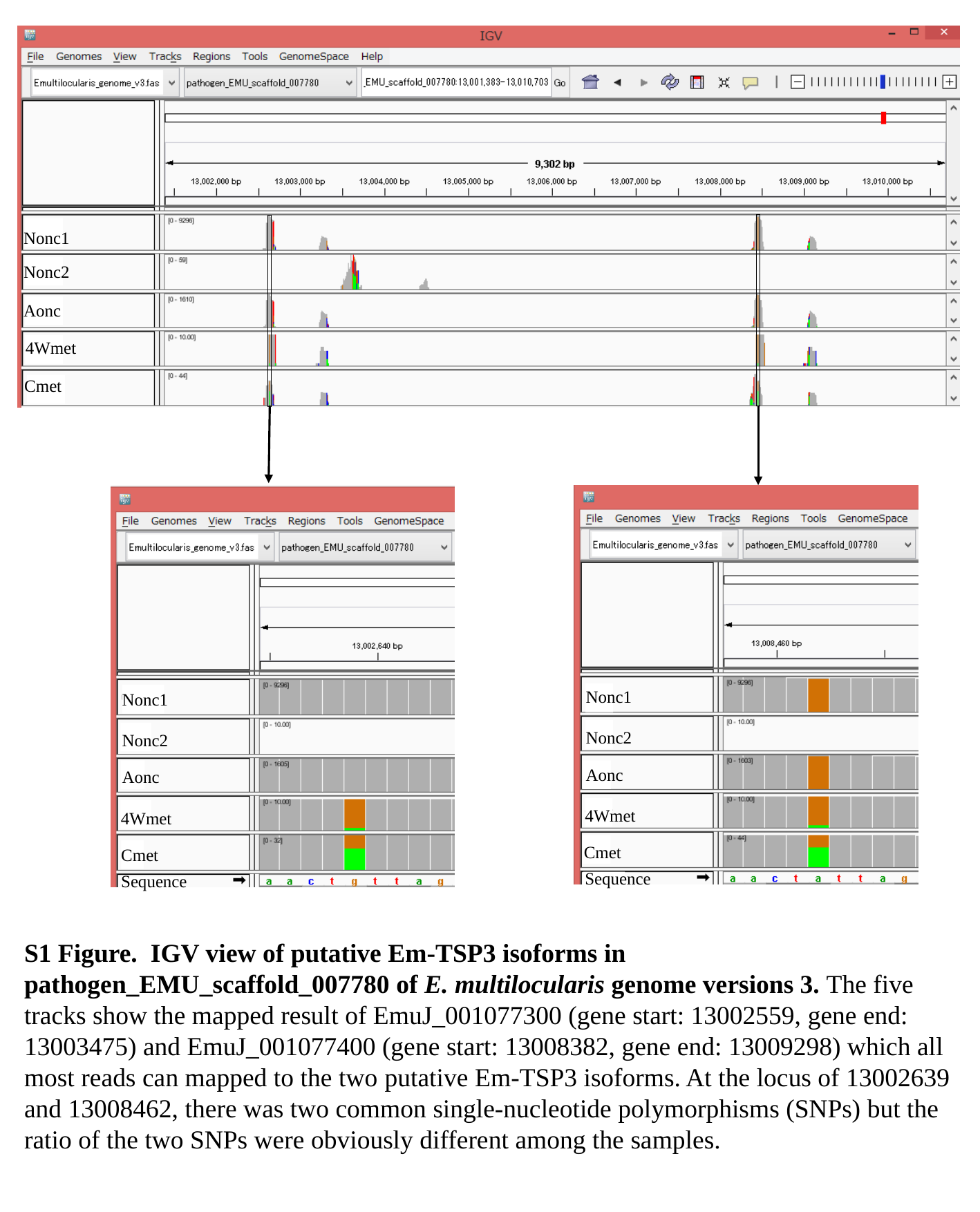

Nonc1
Nonc2
Aonc
4Wmet
Cmet
Nonc1
Nonc1
Nonc2
Nonc2
Aonc
Aonc
4Wmet
4Wmet
Cmet
Cmet
Sequence
Sequence
S1 Figure. IGV view of putative Em-TSP3 isoforms in pathogen_EMU_scaffold_007780 of E. multilocularis genome versions 3. The five tracks show the mapped result of EmuJ_001077300 (gene start: 13002559, gene end: 13003475) and EmuJ_001077400 (gene start: 13008382, gene end: 13009298) which all most reads can mapped to the two putative Em-TSP3 isoforms. At the locus of 13002639 and 13008462, there was two common single-nucleotide polymorphisms (SNPs) but the ratio of the two SNPs were obviously different among the samples.
